# Supplementary material for: Predictors of longitudinal cognitive ageing from age 70 to 82 including APOE e4 status, early-life and lifestyle factors: the Lothian Birth Cohort 1936
Source: Mol Psychiatry. 2022 Dec 8;28(3):1256–71. doi: 10.1038/s41380-022-01900-4 (PMC10005946; doi:10.1038/s41380-022-01900-4)
Supplement: Supplementary file 1 — Supplementary Information [file 41380_2022_1900_MOESM1_ESM.docx]

**Supplementary Information**

**S1 Table.** Number of participants with full and missing data on the covariate variables at baseline age 70

**S2 Table.** Longitudinal cognitive test scores for completers only (N = 431)

**S3 Table.** Cognitive test scores at 70 years and 82 years by *APOE* e4 carrier status

**S4 Table.** Latent growth curve model: unstandardized means and variances for the intercept and slope of each cognitive test (slopes refer to change from age 70 to age 82)

**S5 Table.** Model fit indices for the latent growth curve models

**S6 Table.** Sensitivity analysis (N = 1067); predictors of intercepts (age 70) and slopes of change (age 70 to 82) where predictors are entered simultaneously, excluding participants with self-reported dementia (N = 24) at any point across the follow-up period

**S7 Table.** Sensitivity analysis (N = 1045); predictors of intercepts (age 70) and slopes of change (age 70 to 82) where predictors are entered simultaneously, excluding participants with low MMSE (N = 46) at any point across the follow-up period

**S8 Table.** Sensitivity analysis (N = 688); predictors of intercepts (age 70) and slopes of change (age 70 to 82) where predictors are entered simultaneously, excluding participants (N = 403) who died at any point across the follow-up period

**S9 Table.** Path (SEM) model parameters (standardised coefficients (Estimate), standard errors (SE), and P-values)

**S1 Figure. LBC1936 recruitment and attrition flowchart.** Flowchart showing waves of testing and attrition between waves in the LBC1936 study. Deaths were ascertained using linkage data obtained via the National Health Service Central Register up to April 2021, provided by the National Records of Scotland. Number of deaths (N = 403) is correct at the time of analysis, i.e. after wave 5 assessment, and therefore include deaths among the completers and non-completers.

**S2 Figure.** **Path (SEM) diagram**. Path (SEM) diagram including variables from early-life (age 11 IQ, education, *APOE* e4), mid-life (adult SES) and older age (smoking, alcohol, physical activity, BMI, depressive symptoms, CVD, diabetes, stroke). Age and sex were included in the path model but not shown to reduce visual clutter. All model predictors were regressed on a latent variable of general cognitive function (*g*) intercept and slope, estimated within the model. Single-headed arrows represent regression pathways. Double-headed arrows represent covariances.

**S3 Figure. Raw data regression plots for each cognitive test.** Plots of fitted (non-linear) regression lines through the raw data using ‘loess’ smoothing (ggplot2 in R), normalised for baseline score, to illustrate the differences in trajectories of cognitive change with age by *APOE*e4 carrier status (with shaded 95% confidence intervals). The black line is the regression line for the full sample. Red = non-carrier, blue = carrier.

**S1 Table.** Number of participants with full and missing data on the covariate variables at baseline age 70

|  | **Overall**  **(N = 1,091)** | **Missing** |
| --- | --- | --- |
| **Characteristic** | N | N |
| Age, yrs | 1091 | 0 |
| Education, yrs | 1091 | 0 |
| Age 11 IQ | 1028 | 63 |
| Adult SES | 1070 | 21 |
| Physical activity | 961 | 130 |
| Body mass index | 1089 | 2 |
| Alcohol intake, units | 1091 | 0 |
| Depressive symptoms | 1085 | 5 |
|  |  |  |
| Female | 1091 | 0 |
| Lives alone | 1079 | 12 |
| Current smoker | 1091 | 0 |
| *APOE* e4 carrier | 1028 | 63 |
| CVD | 1091 | 0 |
| Diabetes | 1091 | 0 |
| Stroke | 1091 | 0 |

**S2 Table**. Longitudinal cognitive test scores for completers only (N = 431)

|  | **70 years** | | **73 years** | | **76 years** | | **79 years** | | **82 years** | |
| --- | --- | --- | --- | --- | --- | --- | --- | --- | --- | --- |
| **Cognitive test** | N | M (SD) | N | M (SD) | N | M (SD) | N | M (SD) | N | M (SD) |
| Block Design | 429 | 36.0 (10.0) | 430 | 35.3 (10.3) | 423 | 33.5 (9.9) | 415 | 32.5 (9.5) | 420 | 29.9 (9.6) |
| Matrix Reasoning | 430 | 14.7 (5.0) | 430 | 14.2 (4.9) | 423 | 13.8 (4.9) | 419 | 13.4 (5.0) | 418 | 12.9 (5.2) |
| Spatial Span | 428 | 7.6 (1.4) | 428 | 7.5 (1.3) | 421 | 7.5 (1.4) | 419 | 7.2 (1.4) | 421 | 6.9 (1.4) |
|  |  |  |  |  |  |  |  |  |  |  |
| Digit-symbol Coding | 429 | 60.0 (12.0) | 429 | 59.7 (11.6) | 418 | 57.1 (11.8) | 412 | 53.4 (12.0) | 418 | 51.0 (12.8) |
| Symbol Search | 430 | 25.9 (6.6) | 429 | 25.9 (5.8) | 421 | 25.9 (6.1) | 414 | 23.6 (6.2) | 415 | 22.2 (6.9) |
| Choice Reaction Time (secs) | 430 | 0.622 (0.076) | 430 | 0.633 (0.077) | 420 | 0.663 (0.049) | 419 | 0.691 (0.10) | 423 | 0.722 (0.120) |
| Inspection Time | 423 | 114.1 (10.0) | 425 | 112.8 (11.5) | 411 | 111.5 (11.7) | 375 | 108.7 (11.8) | 382 | 106.0 (12.7) |
|  |  |  |  |  |  |  |  |  |  |  |
| Logical Memory | 431 | 74.6 (17.2) | 431 | 76.9 (17.0) | 423 | 77.1 (17.2) | 419 | 75.7 (18.2) | 421 | 72.1 (21.5) |
| Verbal Pairs | 423 | 28.2 (8.3) | 423 | 28.8 (8.8) | 408 | 28.0 (8.8) | 397 | 28.2 (9.0) | 380 | 27.4 (9.5) |
| Digits Backwards | 431 | 8.1 (2.4) | 431 | 8.2 (2.3) | 425 | 8.1 (2.4) | 422 | 7.7 (2.2) | 426 | 7.2 (2.3) |
|  |  |  |  |  |  |  |  |  |  |  |
| NART | 430 | 35.7 (7.8) | 429 | 35.3 (7.7) | 425 | 35.9 (7.7) | 420 | 36.0 (8.0) | 426 | 36.0 (7.8) |
| WTAR | 430 | 42.2 (6.7) | 429 | 42.0 (6.4) | 425 | 41.8 (6.7) | 420 | 42.0 (6.7) | 426 | 42.2 (6.6) |
| Verbal Fluency | 431 | 43.6 (12.5) | 431 | 44.5 (12.3) | 425 | 44.0 (12.2) | 420 | 44.9 (12.4) | 426 | 44.6 (12.7) |

Note: NART, National Adult Reading Test; WTAR, Wechsler Test of Adult Reading

All tests are positively scored (i.e. higher scores = better performance) with the exception of Choice Reaction Time (in seconds) which is negatively scored (i.e. higher scores = slower performance).

**S3 Table**. Cognitive test scores at 70 years and 82 years by *APOE* e4 carrier status

|  | **70 years** | | | | | | **82 years** | | | | | |
| --- | --- | --- | --- | --- | --- | --- | --- | --- | --- | --- | --- | --- |
|  | ***APOE* e4 carrier**  **(N = 306)** | | ***APOE* e4 non-carrier**  **(N = 722)** | |  | | ***APOE* e4 carrier**  **(N = 113)** | | ***APOE* e4 non-carrier**  **(N = 295)** | |  | |
| **Cognitive test** | N | M (SD) | N | M (SD) | P-value | Cohens d | N | M (SD) | N | M (SD) | P-value | Cohen’s d |
| Block Design | 304 | 33.1 (10.4) | 719 | 34.0 (10.2) | 0.22 | 0.087 | 111 | 27.7 (9.5) | 286 | 30.4 (9.6) | 0.010 | 0.283 |
| Matrix Reasoning | 304 | 12.9 (5.0) | 720 | 13.7 (5.1) | 0.02 | 0.158 | 110 | 11.4 (5.0) | 286 | 13.5 (5.2) | 0.001 | 0.411 |
| Spatial Span | 303 | 7.2 (1.4) | 719 | 7.4 (1.4) | 0.03 | 0.143 | 111 | 6.7 (1.5) | 287 | 7.0 (1.4) | 0.02 | 0.207 |
|  |  |  |  |  |  |  |  |  |  |  |  |  |
| Digit-symbol Coding | 306 | 55.7 (12.6) | 717 | 57.0 (13.1) | 0.14 | 0.101 | 112 | 47.7 (13.1) | 284 | 52.2 (12.5) | 0.001 | 0.351 |
| Symbol Search | 305 | 23.9 (6.8) | 719 | 25.1 (6.2) | 0.08 | 0.184 | 110 | 20.2 (7.6) | 283 | 22.9 (6.6) | 0.001 | 0.379 |
| Choice Reaction Time | 305 | 0.647 (0.088) | 717 | 0.642 (0.086) | 0.35 | 0.057 | 110 | 0.753 (0.138) | 290 | 0.715 (0.113) | 0.004 | 0.301 |
| Inspection Time | 291 | 110.9 (12.1) | 692 | 112.6 (10.5) | 0.03 | 0.150 | 94 | 104.5 (13.2) | 266 | 106.9 (12.4) | 0.07 | 0.187 |
|  |  |  |  |  |  |  |  |  |  |  |  |  |
| Logical Memory | 306 | 70.4 (18.1) | 720 | 71.8 (17.9) | 0.25 | 0.078 | 112 | 66.1 (24.6) | 287 | 74.5 (20.2) | 0.001 | 0.387 |
| Verbal Pairs | 290 | 26.2 (8.8) | 700 | 26.4 (9.3) | 0.83 | 0.022 | 98 | 24.7 (10.5) | 260 | 28.4 (9.1) | 0.001 | 0.377 |
| Digits Backwards | 306 | 7.6 (2.1) | 721 | 7.8 (2.3) | 0.45 | 0.091 | 113 | 6.8 (2.3) | 290 | 7.3 (2.3) | 0.03 | 0.217 |
|  |  |  |  |  |  |  |  |  |  |  |  |  |
| NART | 306 | 34.5 (8.6) | 720 | 34.4 (8.1) | 0.79 | 0.012 | 111 | 35.4 (8.8) | 292 | 36.2 (7.5) | 0.39 | 0.098 |
| WTAR | 306 | 40.8 (7.5) | 720 | 41.0 (7.1) | 0.66 | 0.027 | 111 | 41.5 (7.7) | 292 | 42.5 (6.2) | 0.19 | 0.143 |
| Verbal Fluency | 305 | 42.8 (12.4) | 719 | 42.4 (12.6) | 0.61 | 0.032 | 110 | 43.2 (12.6) | 293 | 43.8 (12.7) | 0.74 | 0.047 |

Note: NART, National Adult Reading Test; WTAR, Wechsler Test of Adult Reading

All tests are positively scored (i.e. higher scores = better performance) with the exception of Choice Reaction Time which is negatively scored (i.e. higher scores = slower performance).

Note: P-values obtained from one-way ANOVA

**S4 Table.** Latent growth curve model: unstandardized means and variances for the intercept and slope of each cognitive test (slopes refer to change from age 70 to age 82)

|  |  | **Intercepts** | | **Slopes** | | **SD change in each test** | |
| --- | --- | --- | --- | --- | --- | --- | --- |
| **Cognitive domain** | **Cognitive test** | Mean (SE) | Variance (SE) | Mean (SE) | Variance (SE) | SD change/yr | Rank order of SD change |
| Visuospatial | Matrix Reasoning | 13.442 (0.146)*** | 17.001 (1.050) | -0.158 (0.014)*** | 0.024 (0.009) | -0.038 | **7** |
|  | Block Design | 34.107 (0.306)*** | 84.482 (4.441) | -0.510 (0.025)*** | 0.142 (0.028) | -0.055 | **5** |
|  | Spatial Span | 7.410 (0.040)*** | 1.114 (0.079) | -0.056 (0.004)*** | 0.001 (0.001) | -0.053 | **6** |
| Processing speed | Symbol Search | 25.090 (0.184)*** | 26.824 (1.619) | -0.372 (0.020)*** | 0.093 (0.016) | -0.072 | **4** |
|  | Digit-Symbol Coding | 57.336 (0.386)*** | 140.788 (6.925) | -0.901 (0.034)*** | 0.453 (0.047) | -0.076 | **3** |
|  | Choice Reaction Time | -6.372 (0.026)*** | 0.569 (0.032) | -0.091 (0.003)*** | 0.004 (0.000) | -0.120 | **1** |
|  | Inspection Time | 112.403 (0.329)*** | 76.675 (5.324) | -0.792 (0.044)*** | 0.392 (0.081) | -0.090 | **2** |
| Memory | Logical Memory | 72.918 (0.535)*** | 236.560 (13.479) | -0.413 (0.069)*** | 1.983 (0.188) | -0.027 | **10** |
|  | Verbal Paired Associates | 26.600 (0.276)*** | 65.336 (3.700) | -0.263 (0.032)*** | 0.363 (0.041) | -0.033 | **9** |
|  | Digit Span Backwards | 7.839 (0.066)*** | 3.376 (0.208) | -0.070 (0.006)*** | 0.002 (0.002) | -0.038 | **8** |
| Verbal ability | NART | 34.301 (0.246)*** | 62.450 (2.811) | 0.001 (0.011) | 0.018 (0.005) | 0.0001 | **13** |
|  | WTAR | 40.892 (0.213)*** | 46.994 (2.133) | -0.024 (0.009)* | 0.011 (0.003) | -0.004 | **12** |
|  | Verbal Fluency | 42.656 (0.374)*** | 131.548 (6.609) | -0.116 (0.031)*** | 0.267 (0.041) | -0.010 | 11 |

Note: SE, standard error; NART, National Adult Reading Test; WTAR, Wechsler Test of Adult Reading; CFI, Comparative Fit Index; TLI, Tucker-Lewis Index; RMSEA, Root Mean Square Error of

Approximation

All values from the baseline multivariate model in which all levels and slope covariances were freely estimated

Choice Reaction Time was multiplied by -10 so that higher scores indicated better performance

Path weights for calculation of the slope factor: Baseline=0; to w2=2.98; to w3=6.75; to w4=9.81; to w5=12.53

SD change/yr is the slope mean divided by the intercept standard deviation; rank order of SD change is from highest (1=most change) to lowest (13=least change)

*p < 0.05; **p < 0.01; ***p < 0.001

**S5 Table.** Model fit indices for the latent growth curve models

| Table | Model type | TLI | CFI | RMSEA |
| --- | --- | --- | --- | --- |
| *Table 3* |  |  |  |  |
| Cognitive tests model | Unadjusted | 0.969 | 0.963 | 0.027 |
|  |  |  |  |  |
| *Table 4* |  |  |  |  |
| Visuospatial | Unadjusted | 0.990 | 0.989 | 0.025 |
| Processing speed | Unadjusted | 0.954 | 0.952 | 0.050 |
| Memory | Unadjusted | 0.958 | 0.955 | 0.053 |
| Verbal ability | Unadjusted | 0.988 | 0.988 | 0.042 |
| General cognitive function | Unadjusted | 0.949 | 0.948 | 0.032 |
|  |  |  |  |  |
| *Table 5* |  |  |  |  |
| Visuospatial | Fully adjusted | 0.989 | 0.977 | 0.022 |
| Processing speed | Fully adjusted | 0.937 | 0.932 | 0.038 |
| Memory | Fully adjusted | 0.942 | 0.935 | 0.037 |
| Verbal ability | Fully adjusted | 0.989 | 0.988 | 0.025 |
| General cognitive function | Fully adjusted | 0.937 | 0.936 | 0.030 |

Note: CFI, Comparative Fit Index; TLI, Tucker-Lewis Index; RMSEA, Root Mean Square Error of Approximation.

**S6 Table.** Sensitivity analysis (N = 1067); predictors of intercepts (age 70) and slopes of change (age 70 to 82) where predictors are entered simultaneously, excluding participants with self-reported dementia (N = 24) at any point across the follow-up period

| **Growth parameters** | **Visuospatial ability** | | **Processing speed** | | **Memory** | | **Verbal ability** | | **General cognitive function** | |
| --- | --- | --- | --- | --- | --- | --- | --- | --- | --- | --- |
|  | Estimate (SE) | P values | Estimate (SE) | P values | Estimate (SE) | P values | Estimate (SE) | P values | Estimate (SE) | P values |
| Intercept on |  |  |  |  |  |  |  |  |  |  |
| Age^—^ | **-0.109 (0.027)** | **<0.001** | **-0.151 (0.027)** | **<0.001** | **-0.157 (0.031)** | **<0.001** | **-0.088 (0.020)** | **<0.001** | **-0.138 (0.021)** | **<0.001** |
| Sex | **-0.266 (0.029)** | **<0.001** | -0.028 (0.031) | 0.37 | **0.125 (0.034)** | **<0.001** | 0.002 (0.022) | 0.93 | -0.046 (0.024) | 0.05 |
| Age 11 IQ^+^ | **0.490 (0.028)** | **<0.001** | **0.440 (0.030)** | **<0.001** | **0.560 (0.033)** | **<0.001** | **0.572 (0.020)** | **<0.001** | **0.670 (0.020)** | **<0.001** |
| Education^+^ | **0.110 (0.032)** | **0.001** | 0.037 (0.033) | 0.26 | **0.154 (0.036)** | **<0.001** | **0.235 (0.023)** | **<0.001** | **0.195 (0.025)** | **<0.001** |
| Adult SES^—^ | **-0.127 (0.032)** | **<0.001** | **-0.136 (0.033)** | **<0.001** | 0.019 (0.036) | 0.61 | **-0.113 (0.024)** | **<0.001** | **-0.122 (0.025)** | **<0.001** |
| Lives alone^—^ | 0.029 (0.028) | 0.30 | -0.005 (0.028) | 0.87 | 0.028 (0.032) | 0.37 | -0.033 (0.021) | 0.12 | -0.007 (0.022) | 0.76 |
| Smoking category^—^ | -0.067 (0.028) | 0.02 | **-0.098 (0.028)** | **0.001** | 0.003 (0.032) | 0.93 | 0.034 (0.021) | 0.10 | -0.025 (0.022) | 0.25 |
| Physical activity^+^ | 0.043 (0.030) | 0.16 | 0.076 (0.032) | 0.02 | 0.041 (0.035) | 0.24 | -0.009 (0.023) | 0.70 | 0.033 (0.024) | 0.18 |
| Body mass index^—^ | **0.079 (0.029)** | **0.005** | 0.049 (0.029) | 0.09 | 0.063 (0.032) | 0.049 | **-0.056 (0.021)** | **0.008** | 0.010 (0.022) | 0.66 |
| Alcohol units, week^+^ | -0.004 (0.029) | 0.88 | -0.021 (0.029) | 0.48 | 0.034 (0.033) | 0.30 | -0.021 (0.021) | 0.34 | -0.016 (0.022) | 0.48 |
| *APOE* e4^—^ | **-0.095 (0.028)** | **0.001** | **-0.091 (0.028)** | **0.001** | -0.019 (0.031) | 0.56 | 0.004 (0.021) | 0.84 | -0.045 (0.022) | 0.04 |
| Depressive symptoms^—^ | -0.059 (0.028) | 0.03 | **-0.106 (0.029)** | **<0.001** | -0.068 (0.032) | 0.03 | -0.022 (0.021) | 0.28 | **-0.067 (0.021)** | **0.002** |
| CVD^—^ | -0.035 (0.028) | 0.20 | **-0.078 (0.028)** | **0.006** | 0.051 (0.031) | 0.11 | 0.024 (0.022) | 0.24 | 0.001 (0.021) | 0.96 |
| Diabetes^—^ | -0.062 (0.028) | 0.03 | -0.065 (0.028) | 0.02 | 0.010 (0.032) | 0.76 | -0.048 (0.021) | 0.02 | -0.053 (0.021) | 0.01 |
| Stroke^—^ | -0.022 (0.028) | 0.42 | **-0.073 (0.028)** | **0.009** | 0.049 (0.031) | 0.12 | 0.027 (0.021) | 0.18 | 0.002 (0.021) | 0.94 |
| Slope on |  |  |  |  |  |  |  |  |  |  |
| Age^—^ | 0.100 (0.074) | 0.15 | 0.055 (0.070) | 0.43 | 0.028 (0.046) | 0.54 | **0.288 (0.071)** | **<0.001** | 0.071 (0.043) | 0.10 |
| Sex | 0.055 (0.069) | 0.43 | 0.134 (0.057) | 0.02 | 0.052 (0.050) | 0.30 | 0.102 (0.068) | 0.13 | 0.089 (0.047) | 0.06 |
| Age 11 IQ^+^ | -0.298 (0.125) | 0.02 | -0.028 (0.070) | 0.69 | -0.020 (0.053) | 0.70 | 0.106 (0.071) | 0.14 | -0.053 (0.049) | 0.28 |
| Education^+^ | -0.106 (0.079) | 0.18 | 0.019 (0.070) | 0.78 | -0.026 (0.053) | 0.62 | -0.167 (0.075) | 0.03 | -0.055 (0.050) | 0.27 |
| Adult SES^—^ | -0.067 (0.078) | 0.39 | 0.043 (0.070) | 0.54 | -0.050 (0.053) | 0.44 | -0.018 (0.073) | 0.81 | -0.004 (0.050) | 0.93 |
| Lives alone^—^ | -0.093 (0.071) | 0.19 | -0.017 (0.050) | 0.74 | -0.040 (0.047) | 0.40 | 0.043 (0.064) | 0.49 | -0.011 (0.044) | 0.80 |
| Smoking category^—^ | -0.138 (0.083) | 0.10 | 0.021 (0.053) | 0.69 | 0.043 (0.051) | 0.40 | **-0.202 (0.074)** | **0.006** | -0.029 (0.047) | 0.55 |
| Physical activity^+^ | 0.044 (0.077) | 0.57 | 0.041 (0.080) | 0.61 | 0.007 (0.052) | 0.90 | 0.055 (0.076) | 0.47 | 0.078 (0.049) | 0.11 |
| Body mass index^—^ | -0.058 (0.071) | 0.42 | -0.058 (0.070) | 0.41 | -0.014 (0.049) | 0.78 | 0.012 (0.065) | 0.85 | -0.021 (0.045) | 0.65 |
| Alcohol units, week^+^ | -0.151 (0.091) | 0.10 | 0.022 (0.071) | 0.75 | -0.067 (0.052) | 0.20 | 0.033 (0.073) | 0.66 | -0.023 (0.048) | 0.64 |
| *APOE* e4^—^ | -0.091 (0.072) | 0.21 | **-0.183 (0.054)** | **0.001** | **-0.196 (0.046)** | **<0.001** | -0.032 (0.063) | 0.61 | **-0.218 (0.042)** | **<0.001** |
| Depressive symptoms^—^ | -0.108 (0.074) | 0.14 | -0.078 (0.070) | 0.26 | 0.005 (0.048) | 0.91 | -0.087 (0.064) | 0.18 | -0.083 (0.044) | 0.06 |
| CVD^—^ | -0.034 (0.064) | 0.59 | -0.023 (0.049) | 0.64 | 0.009 (0.048) | 0.84 | -0.053 (0.065) | 0.41 | -0.035 (0.044) | 0.44 |
| Diabetes^—^ | -0.005 (0.071) | 0.95 | -0.045 (0.054) | 0.40 | -0.082 (0.052) | 0.19 | 0.022 (0.074) | 0.77 | -0.046 (0.048) | 0.34 |
| Stroke^—^ | -0.021 (0.072) | 0.77 | 0.088 (0.055) | 0.11 | 0.038 (0.053) | 0.47 | -0.081 (0.073) | 0.27 | 0.053 (0.049) | 0.27 |

Note: SE, standard error; SES, socio-economic status; CVD, cardiovascular disease

Model estimates are fully standardised

Path weights for calculation of the slope factor: Baseline=0; to w2=2.98; to w3=6.75; to w4=9.81; to w5=12.53

Models were run separately for each domain; general cognitive function is based on the intercepts and slopes of the four cognitive domains

Boldtype indicates statistical significance following FDR (false discovery rate) correction

**S7 Table.** Sensitivity analysis (N = 1045); predictors of intercepts (age 70) and slopes of change (age 70 to 82) where predictors are entered simultaneously, excluding participants with low MMSE (N = 46) at any point across the follow-up period

| **Growth parameters** | **Visuospatial ability** | | **Processing speed** | | **Memory** | | **Verbal ability** | | **General cognitive function** | |
| --- | --- | --- | --- | --- | --- | --- | --- | --- | --- | --- |
|  | Estimate (SE) | P values | Estimate (SE) | P values | Estimate (SE) | P values | Estimate (SE) | P values | Estimate (SE) | P values |
| Intercept on |  |  |  |  |  |  |  |  |  |  |
| Age^—^ | **-0.105 (0.028)** | **<0.001** | **-0.149 (0.028)** | **<0.001** | **-0.152 (0.031)** | **<0.001** | **-0.089 (0.021)** | **<0.001** | **-0.134 (0.021)** | **<0.001** |
| Sex | **-0.270 (0.030)** | **<0.001** | -0.035 (0.032) | 0.27 | **0.119 (0.035)** | **0.001** | -0.005 (0.023) | 0.83 | -0.053 (0.024) | 0.03 |
| Age 11 IQ^+^ | **0.481 (0.029)** | **<0.001** | **0.428 (0.030)** | **<0.001** | **0.560 (0.034)** | **<0.001** | **0.553 (0.021)** | **<0.001** | **0.660 (0.020)** | **<0.001** |
| Education^+^ | **0.118 (0.033)** | **<0.001** | 0.041 (0.033) | 0.22 | **0.166 (0.037)** | **<0.001** | **0.246 (0.024)** | **<0.001** | **0.208 (0.025)** | **<0.001** |
| Adult SES^—^ | **-0.111 (0.032)** | **0.001** | **-0.129 (0.033)** | **<0.001** | 0.028 (0.037) | 0.44 | **-0.118 (0.024)** | **<0.001** | **-0.120 (0.025)** | **<0.001** |
| Lives alone^—^ | 0.037 (0.029) | 0.20 | -0.002 (0.029) | 0.94 | 0.034 (0.032) | 0.30 | -0.038 (0.022) | 0.08 | -0.006 (0.022) | 0.78 |
| Smoking category^—^ | -0.071 (0.029) | 0.01 | **-0.112 (0.029)** | **<0.001** | -0.002 (0.032) | 0.95 | 0.029 (0.021) | 0.18 | -0.034 (0.022) | 0.12 |
| Physical activity^+^ | 0.050 (0.031) | 0.11 | **0.084 (0.032)** | **0.008** | 0.063 (0.035) | 0.74 | 0.003 (0.024) | 0.90 | 0.051 (0.024) | 0.04 |
| Body mass index^—^ | **0.091 (0.029)** | **0.002** | 0.052 (0.030) | 0.08 | 0.075 (0.033) | 0.02 | -0.051 (0.022) | 0.02 | 0.018 (0.023) | 0.44 |
| Alcohol units, week^+^ | -0.004 (0.029) | 0.89 | -0.022 (0.030) | 0.46 | 0.032 (0.034) | 0.34 | -0.018 (0.022) | 0.41 | -0.016 (0.023) | 0.47 |
| *APOE* e4^—^ | **-0.104 (0.029)** | **<0.001** | **-0.107 (0.029)** | **<0.001** | -0.035 (0.033) | 0.28 | 0.006 (0.022) | 0.79 | **-0.057 (0.022)** | **0.011** |
| Depressive symptoms^—^ | -0.061 (0.029) | 0.03 | **-0.115 (0.029)** | **<0.001** | -0.066 (0.032) | 0.04 | -0.017 (0.021) | 0.42 | **-0.069 (0.022)** | **0.002** |
| CVD^—^ | -0.029 (0.029) | 0.31 | -0.071 (0.029) | 0.01 | 0.051 (0.032) | 0.11 | -0.017 (0.021) | 0.42 | 0.000 (0.022) | 0.99 |
| Diabetes^—^ | -0.060 (0.029) | 0.04 | -0.061 (0.029) | 0.04 | 0.012 (0.032) | 0.72 | -0.043 (0.021) | 0.04 | -0.050 (0.022) | 0.02 |
| Stroke^—^ | -0.026 (0.028) | 0.36 | **-0.079 (0.029)** | **0.006** | 0.043 (0.032) | 0.18 | 0.026 (0.021) | 0.22 | -0.005 (0.022) | 0.81 |
| Slope on |  |  |  |  |  |  |  |  |  |  |
| Age^—^ | 0.119 (0.069) | 0.09 | 0.051 (0.065) | 0.43 | 0.038 (0.047) | 0.42 | **0.328 (0.081)** | **<0.001** | 0.053 (0.044) | 0.22 |
| Sex | 0.022 (0.072) | 0.76 | 0.081 (0.056) | 0.15 | 0.033 (0.051) | 0.52 | 0.078 (0.072) | 0.28 | 0.042 (0.048) | 0.38 |
| Age 11 IQ^+^ | **-0.312 (0.097)** | **0.001** | -0.039 (0.065) | 0.55 | -0.046 (0.053) | 0.39 | 0.099 (0.074) | 0.18 | -0.076 (0.050) | 0.12 |
| Education^+^ | -0.114 (0.079) | 0.15 | 0.032 (0.065) | 0.63 | -0.016 (0.053) | 0.76 | -0.143 (0.078) | 0.07 | -0.043 (0.050) | 0.39 |
| Adult SES^—^ | -0.079 (0.077) | 0.30 | 0.043 (0.065) | 0.51 | -0.024 (0.053) | 0.65 | -0.017 (0.073) | 0.81 | -0.020 (0.049) | 0.69 |
| Lives alone^—^ | -0.119 (0.072) | 0.10 | -0.032 (0.052) | 0.54 | -0.027 (0.048) | 0.57 | 0.033 (0.067) | 0.62 | -0.022 (0.045) | 0.63 |
| Smoking category^—^ | -0.139 (0.080) | 0.08 | 0.031 (0.065) | 0.63 | 0.046 (0.052) | 0.37 | **-0.165 (0.078)** | **0.03** | -0.012 (0.048) | 0.80 |
| Physical activity^+^ | 0.015 (0.072) | 0.83 | -0.010 (0.065) | 0.87 | -0.006 (0.053) | 0.90 | 0.035 (0.074) | 0.64 | 0.033 (0.049) | 0.50 |
| Body mass index^—^ | -0.088 (0.074) | 0.24 | -0.070 (0.065) | 0.29 | -0.014 (0.049) | 0.78 | 0.012 (0.069) | 0.87 | -0.031 (0.046) | 0.50 |
| Alcohol units, week^+^ | -0.164 (0.083) | 0.049 | 0.010 (0.067) | 0.88 | -0.065 (0.053) | 0.22 | 0.020 (0.077) | 0.80 | -0.029 (0.049) | 0.55 |
| *APOE* e4^—^ | -0.103 (0.070) | 0.14 | **-0.176 (0.053)** | **0.001** | **-0.192 (0.047)** | **<0.001** | -0.023 (0.066) | 0.73 | **-0.216 (0.043)** | **<0.001** |
| Depressive symptoms^—^ | -0.129 (0.072) | 0.07 | -0.072 (0.066) | 0.27 | 0.011 (0.048) | 0.83 | -0.112 (0.069) | 0.10 | -0.083 (0.045) | 0.07 |
| CVD^—^ | -0.086 (0.070) | 0.22 | -0.074 (0.050) | 0.14 | 0.002 (0.048) | 0.96 | -0.069 (0.068) | 0.31 | -0.076 (0.045) | 0.09 |
| Diabetes^—^ | -0.017 (0.077) | 0.83 | -0.085 (0.055) | 0.12 | -0.084 (0.053) | 0.11 | 0.029 (0.077) | 0.71 | -0.075 (0.044) | 0.12 |
| Stroke^—^ | -0.018 (0.078) | 0.82 | 0.096 (0.055) | 0.08 | 0.040 (0.054) | 0.46 | -0.115 (0.078) | 0.14 | 0.061 (0.049) | 0.22 |

Note: SE, standard error; SES, socio-economic status; CVD, cardiovascular disease

Model estimates are fully standardised.

Path weights for calculation of the slope factor: Baseline=0; to w2=2.98; to w3=6.75; to w4=9.81; to w5=12.53

Models were run separately for each domain; general cognitive function is based on the intercepts and slopes of the four cognitive domains

Boldtype indicates statistical significance following FDR (false discovery rate) correction

**S8 Table.** Sensitivity analysis (N = 688); predictors of intercepts (age 70) and slopes of change (age 70 to 82) where predictors are entered simultaneously, excluding participants (N = 403) who died at any point across the follow-up period

| **Growth parameters** | **Visuospatial ability** | | **Processing speed** | | **Memory** | | **Verbal ability** | | **General cognitive function** | |
| --- | --- | --- | --- | --- | --- | --- | --- | --- | --- | --- |
|  | Estimate (SE) | P values | Estimate (SE) | P values | Estimate (SE) | P values | Estimate (SE) | P values | Estimate (SE) | P values |
| Intercept on |  |  |  |  |  |  |  |  |  |  |
| Age^—^ | **-0.099 (0.033)** | **0.003** | **-0.176 (0.035)** | **<0.001** | **-0.109 (0.038)** | **0.004** | **-0.063 (0.025)** | **0.011** | **-0.108 (0.025)** | **<0.001** |
| Sex | **-0.272 (0.035)** | **<0.001** | -0.082 (0.039) | 0.035 | **0.116 (0.041)** | **0.005** | -0.025 (0.027) | 0.35 | -0.064 (0.028) | 0.02 |
| Age 11 IQ^+^ | **0.496 (0.034)** | **<0.001** | **0.456 (0.038)** | **<0.001** | **0.600 (0.040)** | **<0.001** | **0.571 (0.025)** | **<0.001** | **0.679 (0.024)** | **<0.001** |
| Education^+^ | **0.160 (0.039)** | **0.001** | 0.050 (0.043) | 0.24 | **0.197 (0.045)** | **<0.001** | **0.258 (0.029)** | **<0.001** | **0.233 (0.030)** | **<0.001** |
| Adult SES^—^ | **-0.122 (0.039)** | **0.002** | **-0.119 (0.042)** | **0.004** | 0.037 (0.045) | 0.42 | **-0.098 (0.030)** | **0.001** | **-0.101 (0.030)** | **0.001** |
| Lives alone^—^ | -0.012 (0.034) | 0.73 | -0.069 (0.036) | 0.05 | 0.010 (0.039) | 0.79 | -0.053 (0.026) | 0.04 | -0.045 (0.026) | 0.08 |
| Smoking category^—^ | -0.040 (0.034) | 0.24 | -0.088 (0.036) | 0.015 | 0.051 (0.039) | 0.19 | 0.046 (0.025) | 0.07 | -0.001 (0.026) | 0.97 |
| Physical activity^+^ | -0.006 (0.036) | 0.87 | 0.075 (0.039) | 0.05 | 0.058 (0.041) | 0.16 | -0.008 (0.0238 | 0.77 | 0.021 (0.028) | 0.44 |
| Body mass index^—^ | 0.084 (0.034) | 0.01 | 0.055 (0.037) | 0.14 | 0.012 (0.039) | 0.76 | **-0.077 (0.026)** | **0.003** | -0.013 (0.026) | 0.61 |
| Alcohol units, week^+^ | 0.018 (0.036) | 0.61 | -0.005 (0.038) | 0.90 | 0.019 (0.041) | 0.64 | -0.017 (0.027) | 0.52 | -0.003 (0.027) | 0.91 |
| *APOE* e4^—^ | -0.071 (0.034) | 0.04 | -0.056 (0.036) | 0.13 | -0.043 (0.038) | 0.47 | -0.009 (0.026) | 0.72 | -0.024 (0.026) | 0.35 |
| Depressive symptoms^—^ | -0.056 (0.034) | 0.10 | -0.064 (0.037) | 0.08 | -0.062 (0.038) | 0.11 | -0.053 (0.025) | 0.04 | **-0.070 (0.026)** | **0.006** |
| CVD^—^ | -0.020 (0.033) | 0.54 | -0.046 (0.036) | 0.20 | 0.074 (0.038) | 0.05 | 0.013 (0.025) | 0.60 | 0.013 (0.026) | 0.61 |
| Diabetes^—^ | -0.043 (0.034) | 0.20 | **-0.101 (0.036)** | **0.005** | 0.010 (0.039) | 0.81 | -0.036 (0.025) | 0.16 | -0.049 (0.026) | 0.05 |
| Stroke^—^ | -0.063 (0.033) | 0.06 | -0.065 (0.036) | 0.07 | 0.022 (0.038) | 0.57 | 0.018 (0.025) | 0.14 | -0.017 (0.025) | 0.51 |
| Slope on |  |  |  |  |  |  |  |  |  |  |
| Age^—^ | 0.077 (0.066) | 0.25 | 0.029 (0.092) | 0.75 | -0.054 (0.052) | 0.30 | **0.312 (0.148)** | **0.002** | -0.012 (0.050) | 0.81 |
| Sex | 0.016 (0.065) | 0.81 | 0.039 (0.063) | 0.54 | 0.005 (0.056) | 0.93 | 0.121 (0.101) | 0.18 | -0.005 (0.054) | 0.92 |
| Age 11 IQ^+^ | -0.320 (0.126) | 0.011 | -0.021 (0.093) | 0.82 | -0.049 (0.060) | 0.42 | 0.107 (0.105) | 0.25 | -0.076 (0.058) | 0.19 |
| Education^+^ | -0.143 (0.085) | 0.09 | -0.039 (0.093) | 0.68 | -0.006 (0.060) | 0.92 | -0.203 (0.131) | 0.04 | -0.088 (0.059) | 0.13 |
| Adult SES^—^ | -0.092 (0.079) | 0.26 | -0.032 (0.089) | 0.72 | -0.047 (0.060) | 0.43 | 0.044 (0.098) | 0.64 | -0.037 (0.058) | 0.52 |
| Lives alone^—^ | -0.064 (0.065) | 0.33 | -0.018 (0.058) | 0.76 | -0.039 (0.052) | 0.46 | 0.061 (0.087) | 0.45 | -0.021 (0.050) | 0.68 |
| Smoking category^—^ | -0.120 (0.076) | 0.11 | -0.007 (0.059) | 0.90 | -0.054 (0.054) | 0.32 | **-0.294 (0.146)** | **0.002** | -0.068 (0.052) | 0.19 |
| Physical activity^+^ | 0.063 (0.069) | 0.41 | 0.042 (0.099) | 0.67 | -0.031 (0.056) | 0.58 | -0.021 (0.088) | 0.81 | 0.065 (0.053) | 0.22 |
| Body mass index^—^ | -0.064 (0.069) | 0.36 | -0.048 (0.092) | 0.60 | -0.027 (0.054) | 0.61 | 0.035 (0.085) | 0.68 | -0.017 (0.051) | 0.73 |
| Alcohol units, week^+^ | -0.155 (0.083) | 0.06 | 0.005 (0.093) | 0.95 | -0.068 (0.055) | 0.21 | 0.102 (0.096) | 0.26 | -0.055 (0.053) | 0.30 |
| *APOE* e4^—^ | -0.113 (0.074) | 0.13 | **-0.242 (0.070)** | **<0.001** | **-0.232 (0.051)** | **<0.001** | -0.011 (0.082) | 0.90 | **-0.242 (0.048)** | **<0.001** |
| Depressive symptoms^—^ | -0.071 (0.068) | 0.29 | -0.039 (0.092) | 0.67 | 0.036 (0.052) | 0.49 | -0.084 (0.088) | 0.30 | -0.043 (0.050) | 0.39 |
| CVD^—^ | -0.059 (0.064) | 0.36 | -0.028 (0.056) | 0.61 | 0.057 (0.052) | 0.28 | -0.029 (0.084) | 0.72 | -0.019 (0.051) | 0.70 |
| Diabetes^—^ | -0.016 (0.065) | 0.81 | -0.018 (0.059) | 0.76 | -0.111 (0.056) | 0.045 | 0.154 (0.107) | 0.09 | -0.058 (0.053) | 0.27 |
| Stroke^—^ | 0.007 (0.067) | 0.92 | 0.064 (0.061) | 0.29 | 0.028 (0.057) | 0.62 | -0.105 (0.099) | 0.25 | 0.043 (0.054) | 0.43 |

Note: SE, standard error; SES, socio-economic status; CVD, cardiovascular disease

Model estimates are fully standardised

Path weights for calculation of the slope factor: Baseline=0; to w2=2.98; to w3=6.75; to w4=9.81; to w5=12.53

Models were run separately for each domain; general cognitive function is based on the intercepts and slopes of the four cognitive domains

Boldtype indicates statistical significance following FDR (false discovery rate) correction

**S9 Table.** Path (SEM) model parameters (standardised coefficients (Estimate), standard errors (SE), and P-values)

| **Model paths** | **General cognitive function** | | |
| --- | --- | --- | --- |
| **Early-life and mid-life factors** | Estimate | SE | P values |
| Age11 IQ → education | 0.427 | 0.002 | <0.001 |
| Age11 IQ → adult SES | -0.227 | 0.002 | <0.001 |
| Education → adult SES | -0.366 | 0.023 | <0.001 |
| **Early- and mid-life factors to *g* and other predictors** |  |  |  |
| Age11 IQ → *g* intercept | 0.666 | 0.006 | <0.001 |
| Age11 IQ → *g* slope | -0.040 | 0.000 | 0.374 |
| Age11 IQ → smoking | -0.035 | 0.002 | 0.320 |
| Age11 IQ → alcohol | 0.073 | 0.031 | 0.028 |
| Age11 IQ → physical activity | 0.014 | 0.003 | 0.704 |
| Age11 IQ → BMI | -0.097 | 0.010 | 0.005 |
| Age11 IQ → depressive symptoms | -0.077 | 0.005 | 0.028 |
| Age11 IQ → CVD | -0.084 | 0.001 | 0.017 |
| Age11 IQ → diabetes | -0.092 | 0.001 | 0.008 |
| Age11 IQ → stroke | 0.006 | 0.001 | 0.876 |
| Education → *g* intercept | 0.199 | 0.065 | <0.001 |
| Education → *g* slope | -0.050 | 0.006 | 0.275 |
| Education → smoking | -0.071 | 0.021 | 0.046 |
| Education → alcohol | -0.001 | 0.429 | 0.978 |
| Education → physical activity | 0.079 | 0.037 | 0.037 |
| Education → BMI | -0.036 | 0.136 | 0.314 |
| Education → depressive symptoms | -0.042 | 0.071 | 0.246 |
| Education → CVD | -0.002 | 0.014 | 0.957 |
| Education → diabetes | -0.015 | 0.009 | 0.675 |
| Adult SES → *g* intercept | -0.117 | 0.079 | <0.001 |
| Adult SES → *g* slope | -0.028 | 0.007 | 0.546 |
| Adult SES → smoking | 0.050 | 0.026 | 0.156 |
| Adult SES → alcohol | -0.050 | 0.534 | 0.142 |
| Adult SES → physical activity | -0.068 | 0.046 | 0.067 |
| Adult SES → BMI | 0.081 | 0.168 | 0.022 |
| Adult SES → depressive symptoms | 0.034 | 0.088 | 0.342 |
| Adult SES → CVD | -0.044 | 0.017 | 0.216 |
| Adult SES → diabetes | -0.002 | 0.011 | 0.962 |
| Adult SES → stroke | 0.028 | 0.009 | 0.447 |
| *APOE* e4 → *g* intercept | -0.055 | 0.136 | 0.011 |
| *APOE* e4 → *g* slope | 0.240 | 0.014 | <0.001 |
| *APOE* e4 → CVD | 0.040 | 0.029 | 0.192 |
| **All other predictors to g** |  |  |  |
| Age → *g* intercept | -0.140 | 0.061 | <0.001 |
| Age → *g* slope | 0.045 | 0.006 | 0.261 |
| Sex → *g* intercept | -0.040 | 0.132 | 0.086 |
| Sex → *g* slope | 0.040 | 0.012 | 0.345 |
| Smoking → *g* intercept | -0.020 | 0.091 | 0.358 |
| Smoking → *g* slope | -0.053 | 0.009 | 0.217 |
| Alcohol → *g* intercept | -0.009 | 0.004 | 0.667 |
| Alcohol → *g* slope | -0.025 | 0.000 | 0.563 |
| Physical activity → *g* intercept | 0.032 | 0.061 | 0.169 |
| Physical activity → *g* slope | 0.073 | 0.006 | 0.105 |
| BMI → *g* intercept | 0.017 | 0.014 | 0.446 |
| BMI → *g* slope | -0.047 | 0.001 | 0.262 |
| Depressive symptoms → *g* intercept | -0.066 | 0.028 | 0.002 |
| Depressive symptoms → *g* slope | -0.066 | 0.003 | 0.114 |
| CVD → *g* intercept | -0.005 | 0.141 | 0.803 |
| CVD → *g* slope | -0.051 | 0.014 | 0.215 |
| Diabetes → *g* intercept | -0.054 | 0.222 | 0.012 |
| Diabetes → *g* slope | -0.060 | 0.022 | 0.164 |
| Stroke → *g* intercept | 0.004 | 0.279 | 0.853 |
| Stroke → *g* slope | 0.037 | 0.029 | 0.408 |
| **Lifestyle and health factors (covariances)** |  |  |  |
| Smoking ↔ alcohol | 0.099 | 0.272 | 0.001 |
| Smoking ↔ physical activity | -0.110 | 0.024 | 0.001 |
| Smoking ↔ BMI | -0.057 | 0.086 | 0.060 |
| Smoking ↔ depressive symptoms | 0.076 | 0.045 | 0.012 |
| Smoking ↔ CVD | 0.048 | 0.009 | 0.114 |
| Smoking ↔ diabetes | 0.027 | 0.006 | 0.379 |
| Smoking ↔ stroke | 0.079 | 0.004 | 0.010 |
| Alcohol ↔ physical activity | 0.014 | 0.474 | 0.663 |
| Alcohol ↔ BMI | -0.015 | 1.739 | 0.611 |
| Alcohol ↔ depressive symptoms | -0.029 | 0.900 | 0.331 |
| Alcohol ↔ CVD | 0.012 | 0.173 | 0.122 |
| Alcohol ↔ diabetes | -0.047 | 0.088 | 0.190 |
| Alcohol ↔ stroke | -0.040 | 0.088 | 0.190 |
| Physical activity ↔ BMI | -0.164 | 0.155 | <0.001 |
| Physical activity ↔ depressive symptoms | -0.170 | 0.080 | <0.001 |
| Physical activity ↔ CVD | -0.108 | 0.015 | 0.001 |
| Physical activity ↔ diabetes | -0.135 | 0.010 | <0.001 |
| Physical activity ↔ stroke | -0.056 | 0.008 | 0.104 |
| BMI ↔ depressive symptoms | 0.092 | 0.289 | 0.002 |
| BMI ↔ CVD | 0.093 | 0.056 | 0.002 |
| BMI ↔ diabetes | 0.163 | 0.036 | <0.001 |
| BMI ↔ stroke | -0.009 | 0.028 | 0.768 |
| Depressive symptoms ↔ CVD | 0.108 | 0.029 | <0.001 |
| Depressive symptoms ↔ diabetes | 0.095 | 0.018 | 0.002 |
| Depressive symptoms ↔ stroke | 0.103 | 0.015 | 0.001 |
| CVD ↔ diabetes | 0.101 | 0.004 | 0.001 |
| CVD ↔ stroke | 0.034 | 0.003 | 0.263 |
| Diabetes ↔ stroke | 0.130 | 0.002 | <0.001 |

Note: SE, standard error; SES, socio-economic status; g, general cognitive function; CVD, cardiovascular disease

SES is coded negatively, i.e. a lower value denotes a more professional occupational social class

General cognitive function is a latent variable derived from the intercepts and slopes of the four cognitive domains in latent growth curve models

Single-headed arrows represent regression pathways. Double-headed arrows represent covariances. Estimates are standardised beta regression weights

**
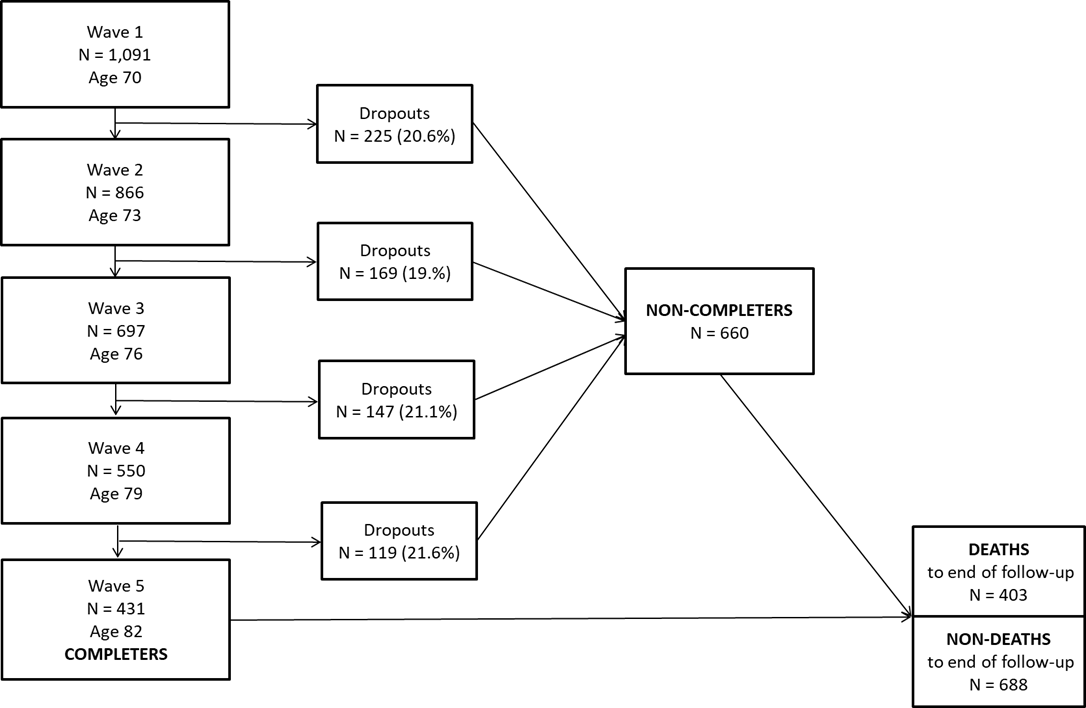
**

**S1 Figure. LBC1936 recruitment and attrition flowchart.** Flowchart showing waves of testing and attrition between waves in the LBC1936 study. Deaths were ascertained using linkage data obtained via the National Health Service Central Register up to April 2021, provided by the National Records of Scotland. Number of deaths (N = 403) is correct at the time of analysis, i.e. after wave 5 assessment, and therefore include deaths among the completers and non-completers.


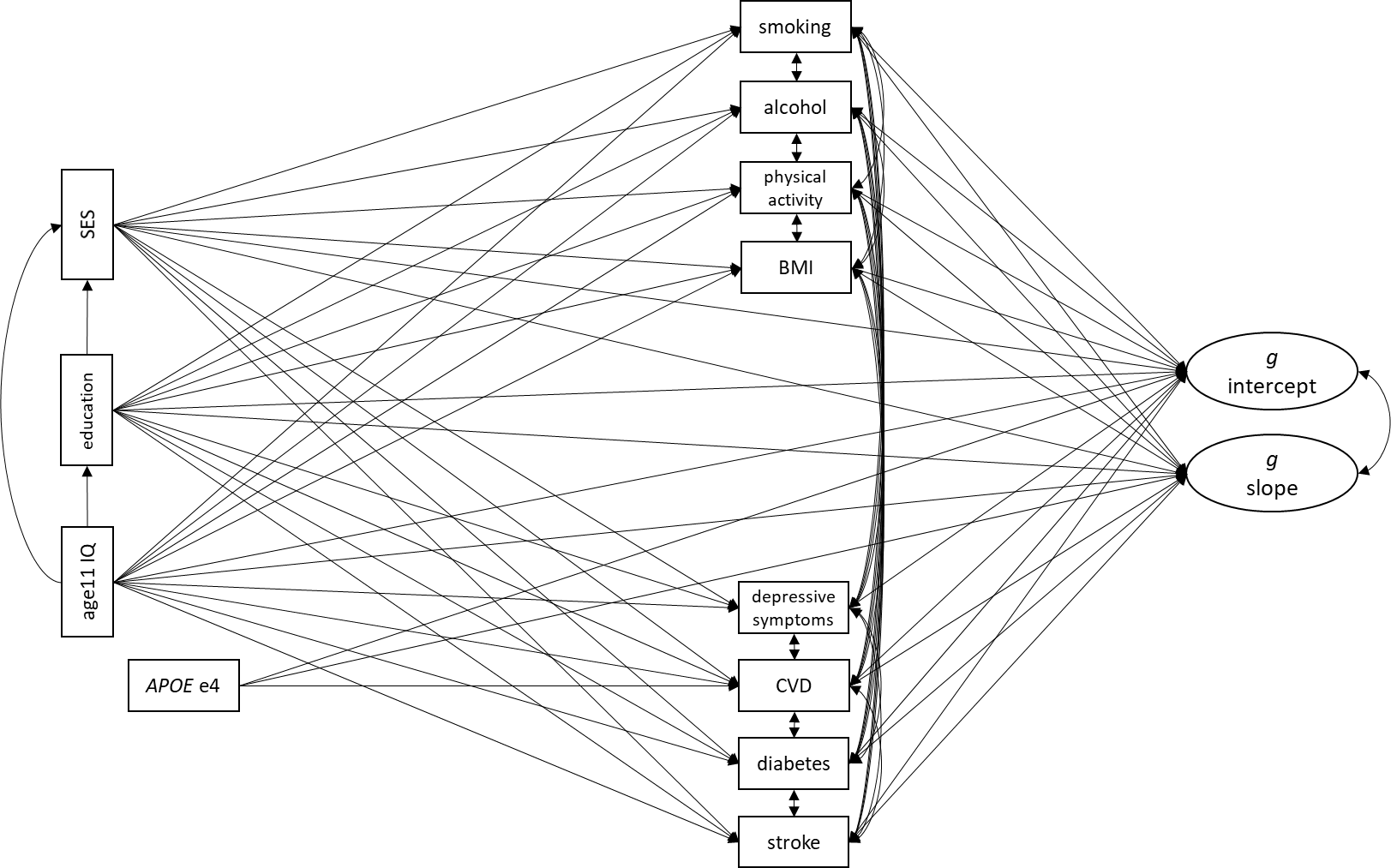


**S2 Figure.** **Path (SEM) diagram**. Path (SEM) diagram including variables from early-life (age 11 IQ, education, *APOE* e4), mid-life (adult SES) and older age (smoking, alcohol, physical activity, BMI, depressive symptoms, CVD, diabetes, stroke). Age and sex were included in the path model but not shown to reduce visual clutter. All model predictors were regressed on a latent variable of general cognitive function (*g*) intercept and slope, estimated within the model. Single-headed arrows represent regression pathways. Double-headed arrows represent covariances.

**
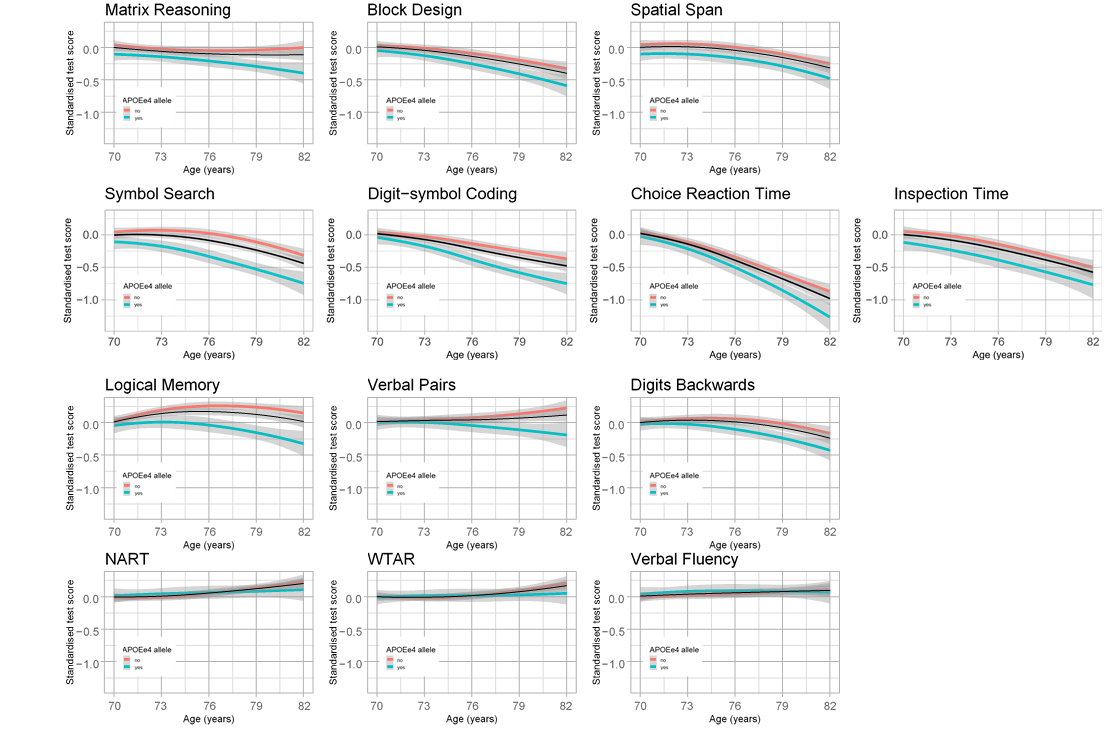
**

**S3 Figure. Raw data regression plots for each cognitive test.** Plots of fitted (non-linear) regression lines through the raw data using ‘loess’ smoothing (ggplot2 in R), normalised for baseline score, to illustrate the differences in trajectories of cognitive change with age by *APOE*e4 carrier status (with shaded 95% confidence intervals). The black line is the regression line for the full sample. Red = non-carrier, blue = carrier.
